# Delamanid-containing regimens over 24 weeks for the treatment of multidrug-resistant/rifampicin-resistant tuberculosis: preliminary results from a single center in a multicenter, prospective, observational study

**DOI:** 10.3389/fmed.2025.1631030

**Published:** 2025-09-29

**Authors:** Fuping Yang, Mingdan Yin, Xingmei Jiang, Xing Zhao, Xuemei An, Yan Liu, Yijia Yuan

**Affiliations:** Department of Tuberculosis, Chongqing Public Health Medical Treatment Center, Chongqing, China

**Keywords:** multidrug-resistant tuberculosis, rifampicin-resistant tuberculosis, delamanid, optimal background regimen, sputum culture conversion

## Abstract

**Background:**

Single center preliminary results from a multicenter, prospective, observational study whose aims were to investigate the efficacy and safety of 24-week delamanid-containing regimens in Chinese multidrug-resistant/rifampicin-resistant tuberculosis (MDR/RR-TB) patients.

**Methods:**

The study included adult patients (≤65 years old) with laboratory-confirmed MDR/RR-TB who were assigned to receive 24 weeks of delamanid (100 mg, twice daily) plus an optimized background regimen (OBR), followed by 5 ~ 12 months of continuation treatment with OBR alone according to World Health Organization and Chinese guidelines.

**Results:**

Thirty-three patients were enrolled, 26 of whom completed the entire treatment course (intensive + continuation phases), with a treatment success rate of 78.8% (95% CI, 61.1, 91.0). Among the 29 patients who were baseline culture-positive, sputum culture conversion was observed in 25 (86.2%) patients within 24 weeks of delamanid treatment, with a median time to sputum culture conversion of 38 days (interquartile range: 24–77). A total of 18 (54.6%) patients experienced treatment-emergent adverse events (TEAEs), most of them being grade 1 or 2 in severity. Six (18.2%) patients had delamanid-related TEAEs, of whom 5 (15.2%) discontinued the delamanid treatment due to QT interval prolongation (2, 6.1%), gastrointestinal reactions (2, 6.1%) or atrial premature beat (1, 3.0%).

**Conclusion:**

The preliminary findings of the present single-center study indicated that the 24-week delamanid-containing regimen demonstrated a promising treatment outcome in Chinese MDR/RR-TB patients. A 24-week follow-up period of safety outcomes was basically consistent with the overall results of the multicenter investigation, and close monitoring of QT interval prolongation should in particular be carried out when delamanid is combined with clofazimine and levofloxacin.

**Clinical trial registration:**

https://clinicaltrials.gov/study/NCT04421495, identifier NCT04421495

## 1 Introduction

Currently, multidrug-resistant and rifampin-resistant tuberculosis (MDR/RR-TB) remains a serious public health problem globally. There were about 400,000 MDR/RR-TB cases worldwide in 2023, with the estimated proportion of new TB cases with MDR/RR-TB falling from 4.0% in 2015 to 3.2% in 2023 (1). China was one of the highest burdened countries with MDR-TB; there were 29,000 new cases of MDR/RR-TB in 2023, with a treatment success rate of 65.89% (50.71% in 2020 ↑ 15.18%), which is still lower than the global success rate of 68.39% in 2021 (evaluated in 2023) (1). Currently, the treatment regimens for MDR/RR-TB include second-line drugs, such as bedaquiline and fluoroquinolone drugs; yet these regimens are costlier than first-line treatment for drug-sensitive TB and give rise to more side effects than first-line treatment. Hence, it has become of paramount importance to develop more effective and safer chemotherapy regimens that will shorten the duration of treatment.

Delamanid was the second new anti-TB drug developed after bedaquiline, and was officially launched in China in 2018. It was included in group C of MDR/RR-TB treatment regimens recommended by the World Health Organization (WHO) and Chinese guidelines in 2019 (2, 3), for the long-term treatment of MDR/RR-TB patients aged ≥3 years old. Delamanid was the first approved nitro-hydro-imidazooxazole derivative with a completely new action mode, acting on the cell wall of *Mycobacterium tuberculosis* and killing the bacterium by inhibiting the synthesis of keto- and methoxy-mycolic acids (4). A phase 3 trial revealed that delamanid was well tolerated when combined with the optimized background regimen (OBR), but failed to demonstrate superior efficacy in shortening sputum culture conversion times (5). Another phase 2/3 non-inferiority trial in South Korea showed that short-term oral delamanid, linezolid, levofloxacin and pyrazinamide regimens had a comparable treatment success rate compared to the conventional treatment regimen, resulting in an earlier sputum culture conversion for fluoroquinolone-sensitive MDR-TB patients (6). However, there is a paucity of relevant research data on delamanid in Chinese MDR/RR-TB patients, most of which were case reports or reports from one of the participating sites in global clinical trials. Consequently, a multicenter, observational study involving 26 research institutions in China was initiated for the first time, with the objective of monitoring and assessing the safety and efficacy of delamanid in combination with OBR for the treatment of MDR/RR-TB (7). The present study reports the preliminary results of a delamanid-containing treatment regimen over 24 weeks in Chinese MDR/RR-TB patients from a single center.

## 2 Materials and methods

### 2.1 Study design and participants

Patients were recruited from the Chongqing Public Health Medical Treatment Center, one of the sites of the single-arm, multicenter, prospective, observational study, which was first launched in July 2020 across 26 centers in China (7). The present study included MDR/RR-TB patients aged 18 to 65 years who could only form an effective treatment regimen by adding delamanid on the basis of drug susceptibility testing results and previous treatment history, in accordance with the WHO and Chinese guidelines of 2019 (2, 3). The diagnosis of MDR-TB or RR-TB was based on microbiological (sputum culture/smear-positive, drug susceptibility testing) and chest radiography findings, in which MDR-TB refers to TB patients who were resistant to at least both isoniazid and rifampicin based on drug susceptibility testing, while RR-TB refers to those who were only resistant to rifampicin. Other inclusion criteria were: patients had not yet started treatment for MDR-TB, or although they had begun anti-TB treatment, they still needed further intensive treatment; the corrected QT interval (QTcF) recorded on the electrocardiogram was < 450 ms; and patients had no history of respiratory failure, heart failure or clinically significant arrhythmias. Detailed inclusion and exclusion criteria are shown in Supplementary Appendix 1.

The study was conducted in accordance with the Declaration of Helsinki and was approved by the ethics committee of Chongqing Public Health Medical Treatment Center (approval number 2020–044-01-KY). Written informed consent was obtained from all enrolled patients. The study was registered at clinicaltrials.gov (identifier NCT04421495) on June 9, 2020.

### 2.2 Treatment and assessments

All patients included in the intensive phase were treated with delamanid plus the OBR for 24 weeks, in which the recommended dose of delamanid was 100 mg (i.e., 2 tablets) twice daily, postprandial. In the event of a missed dose of delamanid, the missed dose was immediately administered. If the time was approaching the next scheduled dose, there was no need to make up the missed dose. Instead, we proceeded directly to administer the next dose as per the regimen. After 24 weeks, the missed doses were replenished by oral administration at a dosage of 100 mg twice a day. The cumulative dosage of the delamanid was 672 tablets. In principle, delamanid should be combined with at least three drugs that were sensitive to the patient’s isolated strain to form a chemotherapy regimen, according to reliable drug susceptibility testing results. In the absence of reliable drug susceptibility testing results, delamanid was used in combination with at least four other drugs to which the patient’s isolated strain was likely to be sensitive, forming a chemotherapy regimen. Potentially sensitive drugs refer to those that have never been used or have been used for less than 3 months, or the main drugs in a treatment regimen that has been proven effective in previous treatments. Additionally, delamanid should not be added alone to a chemotherapy regimen that has been proven ineffective after clinical application. Therefore, according to WHO and Chinese guidelines (2, 3), an individualized OBR was developed by the investigators, based on prior anti-TB treatment history, baseline drug susceptibility testing results, and tolerance to drugs. The present study selected the following drugs in turn: levofloxacin, linezolid, clofazimine, cycloserine, pyrazinamide, ethambutol, prothionamide, amikacin or capreomycin. In principle, the dosage of OBR medication was determined based on the patient’s weight and detailed recommended dosages are presented in Supplementary Appendix 2. During the delamanid treatment period, only levofloxacin among the fluoroquinolone drugs was chosen; if clinically required, moxifloxacin could be used as a substitute for levofloxacin after the end of the intensive phase of treatment. All occurrences of QTcF prolongation > 60 ms were associated with the concomitant use of delamanid and fluoroquinolones. Thus, when the co-administration of these two classes of drugs was unavoidable for formulating an appropriate treatment regimen for MDR/RR-TB, close monitoring of the QTcF interval was initiated. When the QTcF interval was ≥ 500 ms, delamanid or other drugs (i.e., levofloxacin, clofazimine) that may cause QTcF prolongation were discontinued and the electrocardiography (ECG) was reviewed weekly until the QTcF interval was restored to <450 ms, but the use of delamanid was not resumed.

After 24 weeks of delamanid treatment, the OBR was generally continued. For patients with MDR-TB and pre-XDR-TB who had completed the intensive-phase treatment and whose sputum culture was negative at the end of intensive-phase treatment, cure was defined as the culture being negative for at least 5 consecutive measurements, with each sputum culture interval being ≥4 weeks and sputum smear results in the current month negative. Treatment completion was defined as after completion of the treatment regimen (intensive + continuous treatment phases), without evidence of treatment failure (i.e., chest radiography or clinical symptoms progression, serious drug reaction) or without inadequate bacteriological cure records, such as when there were no 5 consecutive sputum culture results (every 4 weeks) after the end of the intensive-phase treatment. Therefore, the treatment duration of the present study protocol was 11 to 18 months, including 6 months of the intensive phase and 5 to 12 months of the continuous phase.

Sputum smears (direct or concentrated methods), clinical symptoms and weight assessment were performed within 1 week before the first dose of delamanid, at weeks 2 and 4 during treatment and then every 4 weeks until the end of treatment. Sputum samples were cultured using Lowenstein-Jenden medium (solid culture) or the mycobacterial growth indicator tube (MGIT) system (liquid culture) at baseline (i.e., within 1 week or day 1 before the first dose of delamanid), at weeks 2 and 4 during treatment, and then every 4 weeks until the end of treatment. Drug susceptibility tests on *Mycobacterium tuberculosis*-positive isolates were performed at baseline (within 1 week or day 1 before the first dose of delamanid) and again at the end of the intensive phase of treatment when the sputum culture was positive. In the course of subsequent treatment, if there was a positive culture and the interval from the last drug sensitivity test was ≥ 24 weeks, drug susceptibility testing was performed again. For patients with treatment failure, a drug susceptibility test was added on the basis of a positive sample from the last treatment failure. Phenotypic drug susceptibility tests (solid or liquid drug sensitivity, microplate assay) or molecular drug susceptibility tests were used at baseline before treatment, while phenotypic drug susceptibility tests were employed during treatment and the follow-up periods. Chest CT examinations were carried out within 1 week before the first dose of delamanid and every 12 weeks during delamanid treatment until the end of treatment, with additional chest CT examinations carried out when delamanid was discontinued. ECG measurements were made within 1 week before the first dose of delamanid (baseline), once at 2-, 4-, 8-, 12-, 16-, 20-, and 24-weeks during delamanid treatment, with an additional ECG examination every 12 weeks after the discontinuation of delamanid. ECG follow-up was performed weekly when the QTcF interval was ≥ 450 ms and < 500 ms or with QTcF intervals ≥ 60 ms from baseline during delamanid treatment until the QTcF interval returned to < 30 ms from baseline, or the QTcF interval was < 450 ms.

### 2.3 Efficacy outcomes

The efficacy outcomes included a favorable outcome, time to sputum culture conversion and the time to sputum smear conversion over 24 weeks. Treatment outcomes included cure, treatment completed, treatment failure, death, loss to follow-up and non-evaluation (Supplementary Appendix 3), where a favorable outcome was defined as the proportion of patients evaluated as cured and treatment completion (intensive + continuation phases). Initial sputum culture/smear conversion was defined as 2 or more consecutive negative culture/smear results at least 4 weeks apart over 24 weeks treatment of delamanid in patients with a positive sputum culture/smear at baseline. The time of sputum culture/smear conversion was the specimen collection date when the negative sputum culture/smear result was first obtained.

### 2.4 Safety outcomes

Safety outcomes included the incidence of treatment-emergent adverse events (TEAEs), drug-related TEAEs and serious AEs (SAE). The severity of TEAEs was graded according to the Division of AIDS (DAIDS) Table for Grading the Severity of Adult and Pediatric Adverse Events Corrected (ver. 2.1) (8). In addition, clinically significant QT prolongation was defined as a QTcF ≥500 ms or a QTcF change from baseline ≥ 60 ms during the intensive-phase treatment as an event of concern. The related definitions of AEs are presented in Supplementary Appendix 4.

### 2.5 Statistical analysis

All statistical analyses were performed using SAS software (SPSS Inc., Chicago, Ill., USA). Continuous variables are expressed as the median with range (minimum-maximum), and categorical variables as numbers with percentages. All patients who received at least one dose of delamanid were included in the safety set, which was used for analysis of safety outcomes during the 24-week delamanid treatment period. Efficacy outcomes were conducted on the full analysis set (FAS) of patients who received at least one dose of delamanid, where a favorable outcome refers to completion of the entire course of treatment (intensive + continuation phases) or earlier cure, while sputum culture/smear conversion was assessed during the 24-week delamanid treatment period. Two-sided 95% confidence intervals (CIs) for favorable outcomes were calculated using the Clopper-Pearson method. For the times to sputum culture/smear conversion, the Kaplan–Meier nonparametric maximum likelihood method was used for point estimation of the survival function over a predictable time period, and the Greenwood formula was combined with the Delta method to construct a two-sided Wald asymptotic 95% CI on a log (−log) scale. Moreover, the corresponding 95% CIs for the median, 25th percentile and 75th percentile were calculated using the Brookmeyer-Crowley method. Furthermore, a descriptive subgroup analysis of efficacy outcomes was conducted stratified by fluoroquinolone resistance (or not) or the numbers of background drugs administered (4 vs. 5 vs. 6 drugs).

## 3 Results

### 3.1 Baseline characteristics

**Figure 1:**
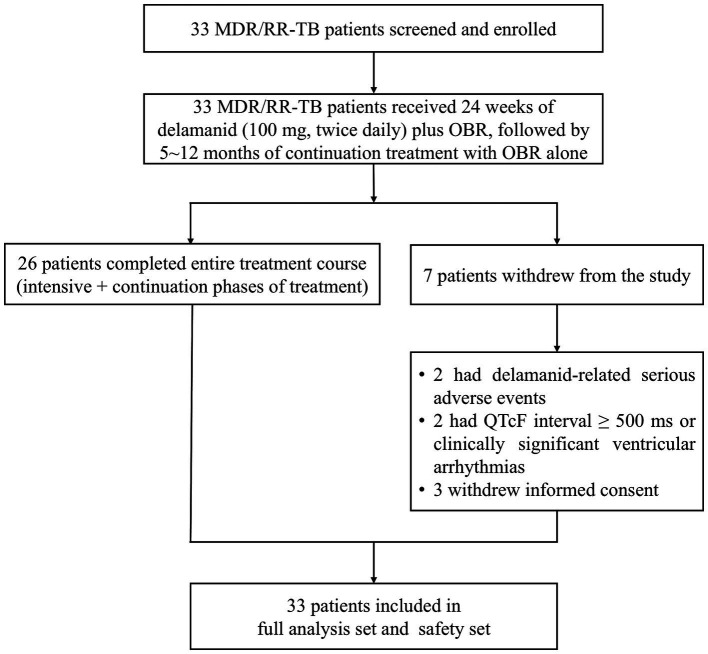
Patient disposition. MDR, multidrug resistant; OBR, optimized background regimen; QTcF, QT interval corrected using Fridericia’s method; RR, rifampicin resistant; TB, tuberculosis.

From September 26, 2020 to October 30, 2021, 33 patients were enrolled in the study to receive delamanid treatment, of whom 26 completed the entire treatment course (intensive + continuation phases). Among the 7 patients who withdrew from the study, 2 had delamanid-related SAEs, 2 had a QTcF interval ≥ 500 ms or clinically significant ventricular arrhythmias, and 3 withdrew their informed consent (Figure 1). Finally, all enrolled 33 patients who received delamanid were included in the FAS and safety set.

The median age of patients was 41 years (range: 22–63), and most were male (20, 60.6%). All enrolled patients had been diagnosed with pulmonary TB, 23 (82.1%) of 28 patients had bilateral involvement, with 19 (67.9%) exhibiting cavities on chest radiographs. There were 8 (24.2%) cases of RR-TB, 15 (45.5%) cases of MDR-TB, and 10 (30.3%) cases of pre-XDR-TB (referred to as tuberculosis caused by *Mycobacterium tuberculosis* that met the definition of MDR/RR-TB and was resistant to any fluoroquinolone) (9). At baseline, 93.5% (29/31) of patients had a positive culture of *Mycobacterium tuberculosis* and 41.4% (12/29) had positive smears. Most of the patients (24, 72.7%) were treated with 5 background drugs, 6 (18.2%) were treated with 4 background drugs and 3 (9.1%) were treated with 6 background drugs. The most commonly used drugs in OBR were cycloserine (97.0%), linezolid (93.9%), clofazimine (93.9%) and levofloxacin (66.7%) (Table 1). The majority of patients (23/29, 79.3%) received delamanid treatment for ≥ 24 weeks, with a median treatment duration of 168 days (range: 28–197).

**Table 1:** Demographic and baseline characteristics of the enrolled patients (FAS).

Items	Delamanid plus OBR (*n* = 33)
Median age (range), years	41 (22–63)
Age category, *n* (%)	
<25 years	3 (9.1)
25–39 years	12 (36.4)
40–65 years	18 (54.5)
Gender, *n* (%)	
Male	20 (60.6)
Female	13 (39.4)
Median BMI (range), kg/m^2^	19.4 (16.4–29.3)
Chest radiograph, *n* (%)	
Bilateral involvement	23/28 (82.1)
Presence of cavity	19/28 (67.9)
Numbers of resistant drugs, *n* (%)	
≤3	21 (63.6)
4–6	7 (21.2)
7–8	5 (15.2)
DST pattern, *n* (%)	
MDR-TB	15 (45.5)
RR-TB	8 (24.2)
Pre-XDR-TB	10 (30.3)
Culture positivity, *n* (%)	29/31 (93.5)
Smear positivity, *n* (%)	12/29 (41.4)
Median number of drugs in background regimen (range)	5 (4–6)
Patients with background drug regimens, *n* (%)	
4	6 (18.2)
5	24 (72.7)
6	3 (9.1)
Drugs in background regimen, *n* (%)	
Cycloserine	32 (97.0)
Linezolid	31 (93.9)
Clofazimine	31 (93.9)
Levofloxacin	22 (66.7)
Pyrazinamide	17 (51.5)
Prothionamide	15 (45.5)
Amikacin or capreomycin	11 (33.3)
Ethambutol	2 (6.1)
Baseline heart rate (beats/min), mean (SD)	85.2 (12.9)
Baseline QTcF interval (ms), mean (SD)	403.9 (14.0)
Previous TB treatment history, *n* (%)	
Initial anti-TB treatment	17 (51.5)
Re-treatment TB	16 (48.5)
Median treatment duration of delamanid (range), days	168 (28–197)
Delamanid exposure, *n* (%)	
<24 weeks	6/29 (20.7)
24 weeks	21/29 (72.4)
>24 weeks	2/29 (6.9)

DST, drug susceptibility test; FAS, full analysis set; MDR, multidrug resistant; QTcF, QT interval corrected using Fridericia’s method; OBR, optimized background regimen; RR, rifampicin resistant; TB, tuberculosis; XDR, extensively drug-resistant.

### 3.2 Efficacy outcomes

A total of 26 patients completed the entire treatment course (intensive + continuous phases), with a treatment success rate of 78.8% (95% CI, 61.1, 91.0) (Table 2). There were 19 fluoroquinolone-sensitive patients and 7 fluoroquinolone-resistant patients who had favorable outcomes, with relatively low treatment success rates in patients who exhibited fluoroquinolone resistance (70.0% vs. 82.6%). In addition, the treatment success rates for patients receiving 4, 5 and 6 background drugs were 83.3% (95% CI, 35.9, 99.6), 75.0% (95% CI, 53.3, 90.2), and 100% (95% CI, 29.2, 100), respectively (Supplementary Table 2).

**Table 2:** Efficacy outcomes of MDR/RR-TB patients.

Efficacy outcomes	Delamanid plus OBR
Treatment outcomes (FAS)	*n* = 33
Favorable outcomes, *n* (%)	26 (78.8)
Cured	0
Treatment completion	26 (78.8)
Unfavorable outcomes, *n* (%)	7 (21.2)
Early discontinuation	7 (21.2)
Adverse events	4 (12.1)
Patient decision	3 (9.1)
Death	0
Treatment success rate (%), 95% CI	78.8 (61.1, 91.0)
Positive sputum culture at baseline (*n* = 29)^*^	
Median days (IQR) to sputum culture conversion over 24 weeks	38.0 (24–77)
Initial sputum culture conversion, n/N (%)	25/29 (86.2)
24-week sputum culture conversion^#^, n/N (%)	12/13 (92.3)
Positive sputum smear at baseline (*n* = 12)^*^	
Median days (IQR) to sputum smear conversion over 24 weeks	18.5 (12–62.5)
Initial sputum smear conversion, n/N (%)	11/12 (91.7)
24-week sputum smear conversion, n/N (%)^#^	5/6 (83.3)

^*^Sputum culture/smear conversion was analyzed based on patients who had positive sputum culture/smear results at baseline. ^#^24-week sputum culture/smear conversion were calculated among the patients with available sputum culture/smear data at 24 weeks. CI, confidence interval; FAS, full analysis set; MDR, multidrug resistant; OBR, optimized background regimen; RR, rifampicin resistant; TB, tuberculosis.

**Figure 2:**
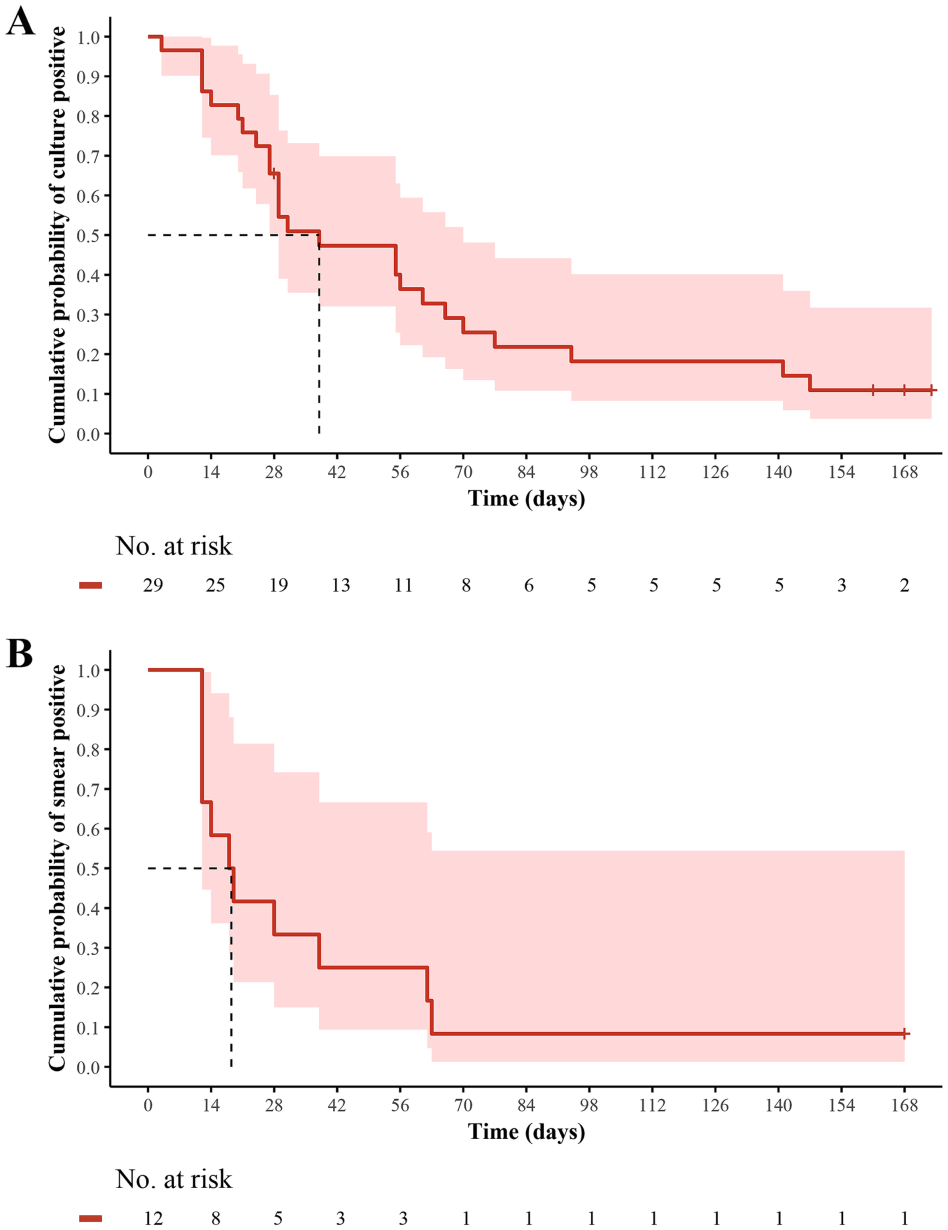
Kaplan–Meier estimates of **(A)** sputum culture conversion and **(B)** sputum smear conversion over 24 weeks in MDR/RR-TB patients who received delamanid plus optimized background regimen. The patients at risk at each time point were those who did not achieve culture conversion and were still participating in the study. MDR, multidrug resistant; RR, rifampicin resistant; TB, tuberculosis.

Two MDR-TB patients had baseline negative culture results that remained negative within 24 weeks. Of 2 RR-TB patients without sputum sample testing at baseline, 1 had a negative culture at 24 weeks, while the other had negative cultures after baseline but turned positive at 24 weeks, and remained negative at subsequent time points and was therefore considered to be a contaminated sample. Among the 29 patients with a baseline culture-positive, the sputum culture conversion was observed in 25 (86.2%) patients within 24 weeks of delamanid treatment, with a median time to sputum culture conversion of 38 days (IQR: 24–77) (Figure 2A). Fluoroquinolone-sensitive patients had a shorter median time to sputum culture conversion (29 vs. 68 days) than fluoroquinolone-resistant patients over 24 weeks of delamanid treatment (Supplementary Table 2). Furthermore, compared with patients receiving 5 or 6 background drugs, those receiving 4 background drugs had the shortest median time to sputum culture conversion (31 vs. 38 vs. 56 days), but the proportion of patients achieving culture conversion within 24 weeks was also the lowest (60.0% vs. 90.5% vs. 100%). At 24 weeks, 12 out of 13 patients (92.3%) had sputum culture conversions, including all 4 fluoroquinolone-resistant patients (100%) and 8 out of 9 fluoroquinolone-sensitive patients (88.9%) (Table 2 and Supplementary Table 2).

Of the 4 patients who did not convert culturally within 24 weeks of delamanid treatment, except for 1 RR-TB patient who withdrew from the study due to QTcF interval prolongation, the remaining 3 patients completed the entire treatment course and achieved favorable outcomes. Two of them had positive culture results at week 4 but no sputum samples were available until week 24; in which, 1 pre-XDR-TB patient converted at week 28, while the other MDR-TB patient still could not produce sputum samples was therefore presumed not to have converted. The remaining 1 patient with MDR-TB had positive sputum cultures at weeks 16, 24 and 32 when sufficient sputum samples were available for testing. However, adequate sputum samples could not be obtained at other time points, and no evidence of treatment failure (clinical symptoms or chest radiography findings progression, or adverse drug reactions) was observed until the end of OBR treatment.

Among the 12 patients with baseline smear-positive results, 11 (91.7%) achieved sputum smear conversion over 24 weeks delamanid treatment, with a median time to sputum smear conversion of 18.5 days (IQR: 12–62.5) (Figure 2B). The median time to sputum smear conversion was similar between patients with fluoroquinolones resistance and those who were sensitive to fluoroquinolones (19 vs. 18 days) (Supplementary Table 2). In addition, the median times to sputum smear conversion for patients receiving 4, 5 and 6 background drugs were 12 (IQR: 12–12), 33 (IQR: 15–62.5) and 16.5 (IQR: 14–19), respectively. At 24 weeks, 5 out of 6 patients (83.3%) had sputum smear conversion, including all 2 fluoroquinolone-resistant patients (100%) and 3 out of 4 fluoroquinolone-sensitive patients (75.0%) (Table 2 and Supplementary Table 2).

### 3.3 Safety outcomes

A total of 18 (54.6%) of 33 patients experienced 32 TEAEs, most of which were of grade 1 or 2 in severity, with 10 patients (30.3%) experiencing grade 3 or 4 AEs (Table 3). The most commonly occurring TEAEs were liver toxicity (6, 18.2%), hematological abnormalities (5, 15.2%), QT interval prolongation (4, 12.1%) and gastrointestinal reactions (4, 12.1%). A total of 6 (18.2%) patients had 7 delamanid-related TEAEs, with the most common being QT interval prolongation (3, 9.1%) and gastrointestinal reactions (2, 6.1%) (Supplementary Table 3). The incidences of SAEs and delamanid-related SAEs were 9.1% and 3.0%, respectively. No patients died during the treatment period and no patients had neuropsychiatric events or mental disorders during the study.

**Table 3:** Safety outcomes of MDR/RR-TB patients (safety set).

Items	Delamanid plus OBR (*n* = 33)	
	Numbers of events	Patients with events, *n* (%)
Any TEAEs	32	18 (54.6)
Grade 1	16	11 (33.3)
Grade 2	4	4 (12.1)
≥Grade 3	12	10 (30.3)
Delamanid-related TEAEs	7	6 (18.2)
Grade 1	3	3 (9.1)
Grade 2	2	2 (6.1)
≥Grade 3	2	2 (6.1)
Any SAEs	3	3 (9.1)
Delamanid-related SAEs	1	1 (3.0)
QTcF interval ≥ 500 ms	1	1 (3.0)
QTcF interval ≥ 60 ms relative to baseline	4	4 (12.1)
At least one QTcF of ≥ 500 ms or had a QTcF of ≥ 60 ms relative to baseline	4	4 (12.1)
TEAEs leading to dose adjustment of delamanid	0	0
TEAEs leading to discontinuation of delamanid treatment	6	5 (15.2)
QT interval prolongation	2	2 (6.1)
Gastrointestinal reaction	3	2 (6.1)
Atrial premature beats	1	1 (3.0)
TEAEs leading to death	0	0

TEAE, treatment-emergent adverse event; MDR, multi-drug resistant; OBR, optimized background regimen; QTcF, QT interval corrected using Fridericia’s method; RR, rifampicin resistant; SAE, serious adverse event; TB, tuberculosis.

Patients treated with delamanid-containing regimens exhibited a significant prolongation of the QTcF interval from baseline for the 24 weeks of treatment (*p* = 0.008; Supplementary Figure 1). The maximum changes from baseline in the QTcF interval was observed at week 16, with a maximum value of 24.1 ms. The mean changes from baseline between weeks 4 and 24 were basically stable, ranging from 20.1 ms to 24.1 ms. Four patients had a QTcF interval ≥ 60 ms relative to baseline or a QTcF interval ≥ 500 ms. Of note, all 4 patients who had QTcF interval prolongation were treated with delamanid, levofloxacin and clofazimine in combination. One case of grade 2 QTcF interval prolongation occurred after 51 days of clofazimine treatment, thought to be related to clofazimine treatment, and the patient recovered after 29 days without dose adjustment or discontinuation. Another case of grade 3 QTcF interval prolongation occurred after 23 days of treatment, which was considered to be related to delamanid, levofloxacin and clofazimine treatment, but the patient recovered after 13 days without dose adjustment or discontinuation. The other 2 cases of QTcF interval prolongation occurred after 28 and 84 days of delamanid treatment, and were considered to be related to the delamanid treatment, resulting in permanent discontinuation of delamanid. Among these 2 patients, 1 case of grade 4 QTcF interval prolongation resulted in hospitalization, which improved after 3 days; another case of grade 1 QTcF interval prolongation improved after 6 days. Furthermore, 2 patients experienced delamanid-related gastrointestinal reactions, which also improved after the permanent discontinuation of delamanid, while 1 patient who presented with atrial premature beats did not improve after the permanent discontinuation of delamanid.

In addition to delamanid, the background regimens also produced TEAEs (Supplementary Table 3). Five patients experienced linezolid-related hematological abnormalities, with the majority (4, 80.0%) being grade 3 or 4 in severity. One patient who experienced 3 episodes of anemia had improved symptoms after 3 dose reductions of linezolid, while the other patient did not improve after dose reductions. One patient had abnormal hemoglobin levels and improved after the discontinuation of linezolid, while the other patient developed anemia and the outcome was unknown after the discontinuation of linezolid. The remaining patient developed 2 episodes of abnormal hemoglobin after 55 days and 68 days of linezolid treatment, respectively, and achieved improvement after the discontinuation of linezolid. Gastrointestinal reactions occurred in 3 patients who were treated with prothionamide, and all patients improved after discontinuation or permanent discontinuation of prothionamide. There were 6 patients with liver toxicity, with 1 case related to the combination of prothionamide and pyrazinamide, 2 cases related to pyrazinamide treatment, and 1 case each associated with levofloxacin or prothionamide, respectively; the other case was associated with chronic hepatitis B.

## 4 Discussion

This single-arm observational study presents the preliminary efficacy and safety outcomes of 33 patients with MDR/RR-TB who received delamanid plus OBR for 24 weeks in a single center. Among them, 8 (24.2%) cases were RR-TB, 15 (45.5%) were MDR-TB, and 10 (30.3%) were pre-XDR-TB. The results demonstrated that 26 patients completed the entire treatment course, with a treatment success rate of 78.8% (95% CI, 61.1, 91.0). Sputum culture and smear conversion within 24 weeks of delamanid treatment were observed in 25 out of 29 patients (86.2%) and in 11 of 12 patients (91.7%), with a median time to sputum culture conversion of 38 days (IQR: 24–77) and a median time to sputum smear conversion of 18.5 days (IQR: 12–62.5), respectively.

Previous studies have shown that the success rate of delamanid-containing regimens was as high as 80.9% in observational studies, and slightly greater than that of the bedaquiline-containing regimens, but, of note, the success rate of bedaquiline-containing regimens was higher in experimental studies (10, 11). Likewise, patients treated with delamanid-containing regimens had a comparable treatment success rate of 78.8% in the present study, which was also consistent with previous Chinese studies (12). The addition of delamanid to OBR improved the culture conversion rate, with culture conversion rates at 24 weeks ranging from 74.0 to 96.6% in previous studies (5, 13–16). In the present study, 86.2% of patients achieved culture conversion within 24 weeks of delamanid treatment and the 24-week culture conversion rate was 92.3%, which was comparable to that in a former phase 3 trial (87.6%) (5). The median time to sputum culture conversion was 38 days (IQR: 24–77) in the present study, which was comparable to the 42 days (IQR: 28–68 days) in patients with baseline positive sputum cultures who received the delamanid-containing regimen in the EndTB observational study (14). A descriptive subgroup analysis showed that fluoroquinolone-sensitive patients had a shorter median time to sputum culture conversion (29 vs. 68 days) than fluoroquinolone-resistant patients over 24 weeks of delamanid treatment. A prospective, single-arm cohort study from India (BEAT-TB) also reported a relatively long time to sputum culture conversion (8.5 weeks) in patients who were fluoroquinolone-resistant and treated with bedaquiline, delamanid, linezolid and clofazimine (17). Similar results were observed in the MDR-END phase 2/3 randomized controlled trial, in which, delamanid was administered orally with linezolid, levofloxacin and pyrazinamide, resulting in earlier sputum culture conversion (24 days on liquid media and 19 days on solid media) in patients with fluoroquinolone-sensitive MDR-TB (6). In the present study, 24 of the 33 patients (72.7%) received concomitant treatment with 5 background drugs, while a greater number of patients (49.2%) received 4 background drugs as opposed to 5 background drugs (38.8%) in a multicenter, prospective, observational study (7). Owing to heterogeneity among MDR/RR-TB patients, the differences in background medications resulting from individualized treatment regimens based on WHO and Chinese guidelines might also have had an impact on the treatment outcomes. Since this was a single-arm study without a comparator group and no head-to-head comparison with previous studies, we only present the data from previous studies that evaluated the efficacy of MDR/RR-TB patients who received the delamanid-containing regimen. Our single-center experience suggested that delamanid plus OBR yielded a satisfactory treatment outcome in MDR/RR-TB, and the efficacy of the delamanid-containing regimen will be further validated in a broader population through a multicenter observational study.

Overall, the delamanid-containing regimen was well tolerated, with 54.6% of patients experiencing 32 TEAEs, most of which were of grade 1 or 2 in severity. The most commonly occurring TEAEs in the present study were liver toxicity, QT interval prolongation, hematological abnormalities and gastrointestinal reactions and similar to those previously reported (18–20). Linezolid has previously been reported to cause hematological abnormalities, most presenting as thrombocytopenia and anemia (20, 21), especially in patients treated with linezolid for more than 2 weeks. Consistent with previous main study findings (7), hematological abnormalities were reported in 5 patients (15.2%), all related to linezolid treatment and were of grade 3 or 4 in the majority of these patients (4, 80.0%). Moreover, 93.9% of the patients received linezolid, which may be the main reason for the relatively higher incidence of hematological abnormalities than found in other studies (5, 12). A prospective study conducted in 3 Belgian centers indicated that a linezolid treatment duration ≥ 10 days and trough concentrations > 8 mg/L were independent risk factors for thrombocytopenia, with high trough concentrations associated with impaired renal function (22). In 1 patient in this study who experienced linezolid-related hematological abnormalities, associated renal toxicity was also found. Therefore, considering the large individual differences in the use of linezolid, it is necessary to do a routine blood test and closely monitor the linezolid plasma concentration in special populations to maintain its efficacy within a certain therapeutic window and to reduce adverse reactions.

Several studies have reported that delamanid may produce QTcF interval prolongation and mental disorders. The concurrent use of multiple drugs was one of the main reasons for QTcF interval prolongation in patients receiving the delamanid-containing regimen, in particular clofazimine and levofloxacin (23, 24). Likewise, all 4 patients (12.1%) who experienced QTcF interval prolongation were treated with delamanid, levofloxacin and clofazimine in combination. One of 4 patients had a QTcF interval prolongation associated only with clofazimine, and 3 others a delamanid-related QTcF interval prolongation, 1 also associated with clofazimine and levofloxacin. In addition, multivariate logistic regression analysis from the main study also revealed that clofazimine was an independent risk factor for QTcF interval prolongation in patients who received a delamanid-containing treatment regimen (7). Among these 4 cases, 2 patients recovered without dose adjustment or discontinuation of delamanid, clofazimine or levofloxacin, and the other 2 patients improved after permanent discontinuation of delamanid. It is worth noting that the occurrence rate of 12.1% with QTcF interval prolongation in Chinese patients was higher than the reported 2.8% and 5.3% found in Korean and South African studies (25, 26), but comparable to the multinational clinical trial (included China) of 9.9% (100 mg twice daily) and 13.1% (200 mg twice daily) in patients receiving the delamanid-containing regimen. Additionally, the incidence of QTcF interval prolongation in Chinese patients receiving bedaquiline treatment was 24.7% (27), which was also higher than the rates of 1.6% and 2.7% among patients in Korea and 16 other countries (25). These results indicating that the QTcF interval prolongation effect of the anti-TB drugs may indicate inter-ethnical differences (28) and that Chinese patients may be more sensitive to novel anti-TB drugs. However, studies on ethnic differences in QTcF prolongation, especially between Chinese and non-Chinese cohorts are scarce, findings that warrant further investigation. Consequently, close ECG monitoring is recommended when using delamanid and other drugs that may prolonged the QTcF interval in clinical practice, and the treatment regimens can be timely adjusted according to the ECG monitoring results and cardiovascular symptoms. Notable delamanid-related psychiatric disorder was not reported in this study, whereas only 7 patients (1.2%) in the multicenter investigation reported a delamanid-related psychiatric disorder. This low rate of psychiatric events may be attributable to spontaneous reporting of patient symptom inquiries rather than to standardized screening, such as questionnaires and psychiatric assessments, and warrants further investigation. In a multinational clinical trial, the incidence of depression was significantly higher with delamanid-containing regimens than with OBR alone (29). The results from global clinical trials in a single center in China also indicated that the combination use of delamanid, isoniazid and levofloxacin resulted in severe paranoia or anxiety and that the symptoms were relieved after discontinuing these drugs (12). Thus, patients with MDR/RR-TB should be closely monitored for symptoms of psychiatric disorder when receiving delamanid treatment, and their treatment regimen accordingly adjusted in a timely manner. Overall, the delamanid-containing regimens were well tolerated over 24 a week treatment period, and the safety outcomes of the single-center study were basically consistent with the overall results of the multicenter investigation.

The present study had a number of limitations: First, it adopted a single-arm design without a comparator group, initially reflecting the single-center results of efficacy and safety in a real-world setting, and laying the foundation for larger-scale pooled analyses. Moreover, some patients had no sputum or insufficient sputum samples to yield culture/smear results, leading to a relatively high rate of missing data at certain time points, which is in line with the characteristics of real-world studies. This finding may be related to insufficient support and management of patients as well as the cautious use of delamanid by clinicians. The individualized OBR used in the present study was developed based on the principles of WHO and Chinese guidelines (2, 3). Considering the complexity of treating MDR/RR-TB, the OBR for each patient was slightly different, which might lead to confounding effects from the background medications other than delamanid when evaluating efficacy and safety profiles. Second, the study had a relatively short follow-up duration and safety data were not assessed for the entire study period, which ended with a 24-week intensive-phase treatment with delamanid plus OBR. Efficacy results were assessed only for treatment outcomes (completed or failure) at the end of the entire study period. Of the 7 patients who did not complete the intensive + continuation phases of treatment, 4 discontinued from the study due to AEs during the 24-week course of delamanid treatment, and the remaining 3 discontinued treatment at their own discretion; thus we failed to capture long-term relapse or late-onset AEs. Additionally, no neuropsychiatric events were reported in this study, possibly because neuropsychiatric events were reported spontaneously by patient symptom inquiry rather than by standardized screening such as questionnaires and psychiatric assessments. Finally, the small sample size limited the statistical power of the efficacy outcomes, especially for the subgroup analyses, which warrants further investigation in a larger cohort of patients. Additionally, predictors for culture conversion (e.g., baseline smear status, cavity presence, and drug resistance patterns) can also be further explored in a larger patient cohort in a multicenter study.

In summary, based on our single-center experience, Chinese MDR/RR-TB patients who received 24 weeks of a delamanid-containing regimen had promising treatment outcomes, with a treatment success rate of 78.8% (95% CI: 61.1, 91.0). Among the 29 patients with baseline culture-positive results, the sputum culture conversion was observed in 25 (86.2%) patients within the 24 weeks of delamanid treatment, with a median time to sputum culture conversion of 38 days (IQR: 24–77). Preliminary findings of our single-center study indicated that the delamanid-containing regimen was generally well tolerated, but close ECG monitoring is particularly recommended when delamanid is combined with levofloxacin and clofazimine, drugs that may significantly prolong the QT interval.

## Data Availability

The original contributions presented in the study are included in the article/Supplementary material, further inquiries can be directed to the corresponding author/s.
