# Supplementary material for: Delamanid-containing regimens over 24 weeks for the treatment of multidrug-resistant/rifampicin-resistant tuberculosis: preliminary results from a single center in a multicenter, prospective, observational study
Source: Front Med (Lausanne). 2025 Sep 29;12:1631030. doi: 10.3389/fmed.2025.1631030 (PMC12515798; doi:10.3389/fmed.2025.1631030)
Supplement: Supplementary file 1 [file Data_Sheet_1.pdf]

# Delamanid-containing regimens over 24 weeks for the treatment of multidrug-resistant/rifampicin-resistant tuberculosis: preliminary results from a single center in a multicenter, prospective, observational study

**Running title: Delamanid-containing regimens for MDR/RR-TB**

**Fuping Yang*, Mingdan Yin, Xingmei Jiang, Xing Zhao, Xuemei An, Yan Liu, Yijia Yuan**

Department of Tuberculosis, Chongqing Public Health Medical Treatment Center, Chongqing, China

***Correspondence:**

Fuping Yang

baoer74@qq.com

*Supplementary Material*

**Supplementary Appendix 1. Inclusion and exclusion criteria**

***1. Inclusion criteria; patients who met all of the following criteria were enrolled:***

1) Patients with multidrug-resistant/rifampicin-resistant pulmonary tuberculosis with laboratory diagnostic evidence;
2) Patients aged ≥ 18 years and ≤ 65 years;
3) Patients who had not yet started treatment for multidrug-resistant tuberculosis, or had already begun treatment but required further intensive therapy;
4) In accordance with the World Health Organization (WHO) and Chinese guidelines, an effective treatment regimen could be formed by adding delamanid according to drug susceptibility testing results and previous treatment history;
5) There was no history of respiratory failure, cardiac dysfunction or clinically significant arrhythmia; and a QTcF < 450 ms on electrocardiography records;
6) Participants should be able to take the medication as prescribed by the study protocol and complete treatment and monitoring during the treatment and follow-up periods, and report any adverse reactions to their responsible physician in a timely manner;
7) Patients had agreed to sign an informed consent form.

***2. Exclusion criteria; patients were excluded from this study if they met any of the following criteria:***

1) Had a history of allergy to nitroimidazole and pyrrole-based drugs such as delamanid, metronidazole, tinidazole or any excipients;
2) Serum aminotransferase ≥ 3 × the upper limit of normal (ULN), total bilirubin ≥ 2.5 × ULN; serum albumin < 2.8 g/dL, or those patients who had severe renal function impairment;
3) Currently taking a drug that was a strong inducer of CYP3A4 (i.e., carbamazepine);
4) A pregnant woman or a woman who may become pregnant during the study;
5) Patients who had participated in clinical trials of other off-market novel drugs within 3 months prior to screening;
6) Patients with known congenital QT interval prolongation or any condition that prolonged the QTcF interval or QTcF ≥ 500 ms;
7) Had a history of symptomatic arrhythmia or clinically significant bradycardia;
8) Any cardiac condition that could induce arrhythmia, such as severe hypertension, left ventricular hypertrophy (including hypertrophic cardiomyopathy), or congestive heart failure with decreased left ventricular ejection fraction;
9) Patients with electrolyte imbalances, especially hypokalemia, hypocalcemia or hypomagnesemia;
10) Currently taking a medication known to prolong the QTcF interval, including but not limited to:
    a) Anti-arrhythmic drugs, such as amiodarone, disopyramide, dofetilide, ibutilide, procainamide, quinidine, hydroquinidine or sotalol;
    b) Antipsychotic drugs, such as phenothiazine, sertindole, sultopride,

chlorpromazine, haloperidol, mesoridazine, pimozide or thioridazine, and antidepressants;

c) Certain antibiotics, such as erythromycin, clarithromycin and other macrolides; moxifloxacin, sparfloxacin, triazole-based antifungal agents, pentamidine or saquinavir;
d) Certain non-sedative antihistamines, such as terfenadine, astemizole and mizolastine.

11) Other cardiovascular risk factors drugs, such as cisapride, droperidol, domperidone, bepridil, diphenmethanil, probucol, levofloxacin, methadone, vinblastine or arsenic trioxide.

**Supplementary Appendix 2. Recommended dosage of drugs in OBR for the enrolled 33 patients**

| Group | Drugs | Daily dose used according to body weight | Dosing according to body weight (mg) | | | | | Maximum daily dose (mg) |
|---|---|---|---|---|---|---|---|---|
| | | | 30-35 kg | 36-45 kg | 46-55 kg | 56-70 kg | > 70 kg | |
| A | Levofloxacin | - | 600 | 750 | 750 | 1000 | 1000 | 1500 |
| | Linezolid | - | Refer to the dose for children aged ≤ 14 years | | 600 | 600 | 600 | 1200 |
| B | Clofazimine | - | 100 | 100 | 100 | 100 | 100 | 100 |
| | Cycloserine | 10-15 mg/kg | 500 | 500 | 750 | 750 | 750 | 1000 |
| C | Pyrazinamide | 20-30 mg/kg | 1000 | 1500 | 1500 | 1500 | 2000 | 2000 |
| | Ethambutol | 15-25 mg/kg | 750 | 750 | 1000 | 1000 | 1250 | 1250 |
| | Prothionamide | 15-20 mg/kg | 500 | 500 | 600 | 600 | 800 | 1000 |
| | Amikacin | 15-20 mg/kg | 400 | 400 | 600 | 600 | 800 | 1000 |
| | Capreomycin | 15-20 mg/kg | 500 | 750 | 750 | 750 | 750 | 1000 |

## Supplementary Appendix 3. Definition of treatment outcomes in efficacy evaluations

After 24 weeks of delamanid treatment, the OBR was generally continued. For patients with MDR-TB and pre-XDR-TB who had completed the intensive-phase treatment and whose sputum culture was negative at the end of intensive-phase treatment, cure can be defined as sputum culture negative for at least 5 consecutive measurements, with each sputum culture interval being $\geq$ 4 weeks, and sputum smear results in the current month was negative. In addition, if there were no three consecutive sputum culture results after the end of the intensive-phase treatment, treatment should be continued until it reaches 18 months, without evidence of treatment failure (i.e. chest radiography progress, clinical symptoms progress), then treatment was considered completed. Therefore, the total treatment course of OBR in the present study was 11 to 18 months.

1. Cure: after the end of the intensive period, there were 5 consecutive negative sputum cultures, with an interval of at least 4 weeks between each one, and no evidence of treatment failure.

2. Treatment completion: after completion of the treatment regimen, no evidence of treatment failure was found, and there were no 5 consecutive negative sputum culture results (with an interval of 4 weeks for each).

3. Treatment failure: During the last 12 months of the treatment course, treatment was discontinued or at least two anti-tuberculosis drugs were permanently replaced for any of the following reasons: 1) following the intensive-phase treatment, the sputum culture test remained positive; 2) bacteriological positivity appeared again in the continuous period after culture conversion; 3) evidence of resistance to the anti-tuberculosis drugs of OBR was discovered during its course; 4) clinical symptoms or chest radiography findings worsened, or adverse drug reactions occurred.

4. Death: Deaths occurring during the course of treatment for various reasons.

5. Lost to follow-up: Patients had remained untreated or experienced treatment interruption for any cause for a consecutive period of more than 2 months during the therapeutic process.

6. Not evaluated: patients were transferred to other medical institutions or the treatment outcomes were unknown.

**Supplementary Appendix 4. Related definitions of treatment-emergent adverse events (TEAEs)**

**1. Definition of serious adverse events (SAEs)**

SAE refer to TEAE that occurred during a clinical trial that required hospitalization, a prolonged hospital stay, caused disability, impaired work ability, threatened life or resulted in death, or led to congenital malformations.

Life-threatening refers to a situation where the patient was at risk of death when the event occurred. It did not refer to a TEAE that was presumed to have caused death, only if it had been more severe.

Any TEAEs that resulted in hospitalization or prolonged hospitalization was considered a SAE unless one of the following exceptions occurred:

1) A length of hospital stays < 12 h;
2) Hospitalization was prearranged (i.e. surgery had been selected or scheduled before the study began)
3) Hospitalization was not associated with adverse reactions (e.g., general hospitalization for respite care, reimbursement of medical expenses).

Disability refers to a patient's substantial impairment of functions.

**2. Definition for severity grading of TEAEs**

Grade 1: Temporary or mild discomfort (< 48 h) which did not necessitate medical intervention or treatment.

Grade 2: Mild to moderate activity limitations, where assistance may be necessary, but not, or only minimal medical intervention or treatment was required.

Grade 3: Obvious activity limitation, typically needed significant assistance, which required medical intervention, and possibly hospitalization.

Grade 4: Severely restricted activity, with an extremely urgent need for assistance, highly necessary medical intervention or treatment, and a significant possibility of requiring hospitalization or convalescence.

Grade 5: Any cause of death.

**3. Definition for association with TEAEs**

The relationship between delamanid and TEAEs was evaluated as definitely related, probable related, possibly related, possibly unrelated, to be evaluated, or not evaluated.

1) Definitely related: the sequence of drug use and reaction occurrence was reasonable; the adverse reaction stopped, rapidly subsided or improved after the discontinuation of drug administration (some TEAEs may have occurred several days after discontinuing the medication, depending on the individual's immune status); upon repeated use, the reaction occurred again and may have become significantly worse (i.e., positive re-stimulation test); and there was supporting literature; and other confounding factors such as the original disease had been excluded.

2) Probable related: there was no history of repeated medication, and the rest was the same as "definitely related", or although there was concomitant medication, the possibility of concomitant medication causing the reaction could be excluded.

3) Possible related: there was a close association between the medication and the occurrence time of TEAEs, which was supported by literature data, but the TEAEs could not be excluded if there was more than one drug or the progression factor of the original disease.

4) Possible unrelated: TEAEs were not closely related to the duration of medication, the reaction manifestations were not consistent with the known TEAEs of the drug, and the original disease may also have led to the development of similar clinical manifestations.

5) To be evaluated: the report lacks complete information, and once the missing information is provided, it will be evaluated. Additionally, the causal relationship was difficult to determine, and there was a lack of supporting literature.

6) Not evaluated: There were too many missing pieces of information and it was difficult to establish causality.

**Supplementary Table 1. Individual resistance patterns and baseline drug susceptibility results of all patients**

| Patient ID | Resistance pattern | Drugs with bacillary resistance | OBR in intensive-phase treatment |
|---|---|---|---|
| 1 | Pre-XDR-TB | H, R, Lfx, Mfx, E, Pto, PAS | Lzd, Cfz, Cs, Z, Pto |
| 2 | MDR-TB | H, R, Am | Lfx, Cfz, Cs, Lzd, Cm |
| 3 | RR-TB | R | Lfx, Cs, Pto, Z |
| 4 | MDR-TB | H, R, PAS | Lfx, Lzd, Cs, Cfz |
| 5 | MDR-TB | H, R, Pto | Lfx, Am, Cs, Lzd, Z, Cfz |
| 6 | MDR-TB | H, R, PAS | Lfx, Lzd, Cfz, Cs, Z |
| 7 | MDR-TB | H, R, | Lfx, Lzd, Cfz, Cs, Z |
| 8 | MDR-TB | H, R, E, PAS | Lfx, Lzd, Cs, Pto, Cm, Cfz |
| 9 | MDR-TB | H, R | Lfx, Lzd, Cfz, Cs, Z |
| 10 | MDR-TB | H, R | Lfx, Lzd, Cfz, Cs, Z |
| 11 | RR-TB | R | Lfx, Lzd, Cfz, Pto, Z |
| 12 | MDR-TB | H, R | Lfx, Lzd, Cs, Pto, Z |
| 13 | MDR-TB | H, R | Lzd, Cfz, Cs, E, Z |
| 14 | Pre-XDR-TB | H, R, Lfx, E, Pto, Am, Cm | Lzd, Cfz, Cs, Pto, Z |
| 15 | Pre-XDR-TB | H, R, Lfx, Mfx, Pto, Am | Lzd, Cfz, Cs, Pto, E |
| 16 | RR-TB | R | Lfx, Cfz, Cs, Pto, Am |
| 17 | Pre-XDR-TB | H, R, Lfx, Mfx, E, PAS | Lzd, Cfz, Cs, Pto, Am |
| 18 | RR-TB | R | Lfx, Lzd, Cfz, Cs, Z |
| 19 | MDR-TB | H, R | Lfx, Lzd, Cfz, Cs, Z |
| 20 | RR-TB | R | Lfx, Lzd, Cfz, Cs |
| 21 | Pre-XDR-TB | H, R, Lfx, Mfx, E | Lzd, Cfz, Cs, Pto, Am |
| 22 | Pre-XDR-TB | H, R Lfx, Mfx | Lzd, Cfz, Am, Cs |
| 23 | Pre-XDR-TB | H, R, Lfx, Mfx, E | Lzd, Cfz, Cs, Am, Z |
| 24 | Pre-XDR-TB | H, R, Lfx, Mfx, PAS | Lzd, Cfz, Cs, Pto, Am |
| 25 | RR-TB | R | Lfx, Lzd, Cfz, Cs |
| 26 | RR-TB | R | Lfx, Lzd, Cs, Cfz |
| 28 | MDR-TB | H, R, E | Lfx, Lzd, Cfz, Cs, Z |
| 29 | MDR-TB | H, R, E | Lzd, Cfz, Cs, Pto, Cm |
| 30 | Pre-XDR-TB | H, R, Lfx, Mfx, Z, E, S | Lfx, Lzd, Cfz, Am, Pto, Cs |
| 31 | RR-TB | R | Lfx, Lzd, Cfz, Cs, Z |
| 32 | Pre-XDR-TB | H, R, Lfx, Mfx, Z, E, PAS | Lzd, Cfz, Cs, Pto, Cm |
| 33 | MDR-TB | H, R, Z, E, Am, Cm, Km, S | Lfx, Lzd, Cfz, Cs, Pto |
| 34 | MDR-TB | H, R | Lfx, Lzd, Cfz, Cs, Z |

Note. Am, amikacin; Cfz, clofazimine; Cm, capreomycin; Cs, cycloserine; E, ethambutol; H, isoniazid; Km: kanamycin; Lfx, levofloxacin; Lzd, linezolid; Mfx: moxifloxacin; PAS: para-aminosalicylic acid; Pto, prothionamide; OBR, optimized background regimen; R: rifampicin; S: streptomycin; Z, pyrazinamide

**Supplementary Table 2. Subgroup analyses for efficacy outcomes**

| | Fluoroquinolone-resistant patients (n = 10) | Fluoroquinolone-sensitive patients (n = 23) | Patients with the following number of background drugs | | |
|---|---|---|---|---|---|
| | | | 4 (n = 6) | 5 (n = 24) | 6 (n = 3) |
| Treatment outcome, n (%) | | | | | |
| Favorable outcomes | 7 (70.0) | 19 (82.6) | 5 (83.3) | 18 (75.0) | 3 (100) |
| Cure | 0 | 0 | 0 | 0 | 0 |
| Treatment completion | 7 (70.0) | 19 (82.6) | 5 (83.3) | 18 (75.0) | 3 (100) |
| Unfavorable outcomes due to early discontinuation | 3 (30) | 4 (17.4) | 1 (16.7) | 6 (25.0) | 0 |
| Adverse events | 2 (20) | 2 (8.7) | 1 (16.7) | 3 (12.5) | 0 |
| Patient decision | 1 (10) | 2 (8.7) | 0 | 3 (12.5) | 0 |
| Treatment success rate (%), 95% CI | 70.0 (34.8, 93.3) | 82.6 (61.2, 95.0) | 83.3 (35.9, 99.6) | 75.0 (53.3, 90.2) | 100 (29.2, 100) |
| Positive sputum culture at baseline (n = 29)* | n = 10 | n = 19 | n = 5 | n = 21 | n = 3 |
| Median days (IQR) to sputum culture conversion over 24 weeks | 68 (24-94) | 29 (21-56) | 31 (29-NA) | 38 (21-77) | 56 (27-66) |
| Initial sputum culture conversion, n/N (%) | 9/10 (90.0) | 16/19 (84.2) | 3/5 (60.0) | 19/21 (90.5) | 3/3 (100) |
| 24-week sputum culture conversion, n/N (%)# | 4/4 (100) | 8/9(88.9) | 3/3 (100) | 7/8 (87.5) | 2/2 (100) |
| Positive sputum smear at baseline (n = 12)* | n = 5 | n = 7 | n = 2 | n = 8 | n = 2 |
| Median days (IQR) to sputum smear conversion over 24 weeks | 19 (12-28) | 18 (12-63) | 12 (12-12) | 33 (15-62.5) | 16.5 (14-19) |
| Initial sputum smear conversion, n/N (%) | 5/5 (100) | 6/7 (85.7) | 2/2 (100) | 7/8 (87.5) | 2/2 (100) |
| 24-week sputum smear conversion, n/N (%)# | 2/2 (100) | 3/4 (75.0) | NA | 3/4 (75.0) | 2/2 (100) |

Note. *Sputum culture/smear conversion was analyzed based on the patients who had a positive sputum culture/smear results at baseline.
#24-week sputum culture/smear conversion were calculated among the patients with available sputum culture/smear data at 24 weeks.
; Abbreviations: IQR, interquartile range; NA, not available; OBR, optimized background regimen.

**Supplementary Table 3. Summary of drug-related TEAEs (safety set)**

| Items | Delamanid plus OBR (n = 33) | |
|---|---|---|
| | **Any grade, n (%)** | **≥ grade 3, n (%)** |
| Delamanid related TEAEs | 6 (18.2) | 2 (6.1) |
| QT interval prolongation | 3 (9.1) | 2 (6.1) |
| Gastrointestinal reaction | 2 (9.1) | 0 |
| Atrial premature beats | 1 (3.0) | 0 |
| Linezolid related TEAEs | 5 (15.2) | 5 (15.2) |
| Hematological abnormalities | 5 (15.2) | 4 (12.1) |
| Renal toxicity | 1 (3.0) | 0 |
| Prothionamide related TEAEs | 5 (15.2) | 1 (3.0) |
| Gastrointestinal reaction | 3 (9.1) | 1 (3.0) |
| Liver toxicity | 2 (6.1) | 0 |
| Pyrazinamide related TEAEs | 3 (9.1) | 2 (6.1) |
| Liver toxicity | 3 (9.1) | 2 (6.1) |
| Renal toxicity | 1 (3.0) | 1 (3.0) |
| Levofloxacin related TEAEs | 2 (6.1) | 1 (3.0) |
| Liver toxicity | 1 (3.0) | 1 (3.0) |
| QT interval prolongation | 1 (3.0) | 0 |
| Clofazimine related TEAEs | 2 (6.1) | 1 (3.0) |
| QT interval prolongation | 2 (6.1) | 1 (3.0) |
| Amikacin related TEAEs | 1 (3.0) | 0 |
| Ototoxicity | 1 (3.0) | 0 |

Abbreviations: TEAE, treatment-emergent adverse event; MDR, multidrug resistant; OBR, optimal background regimen.

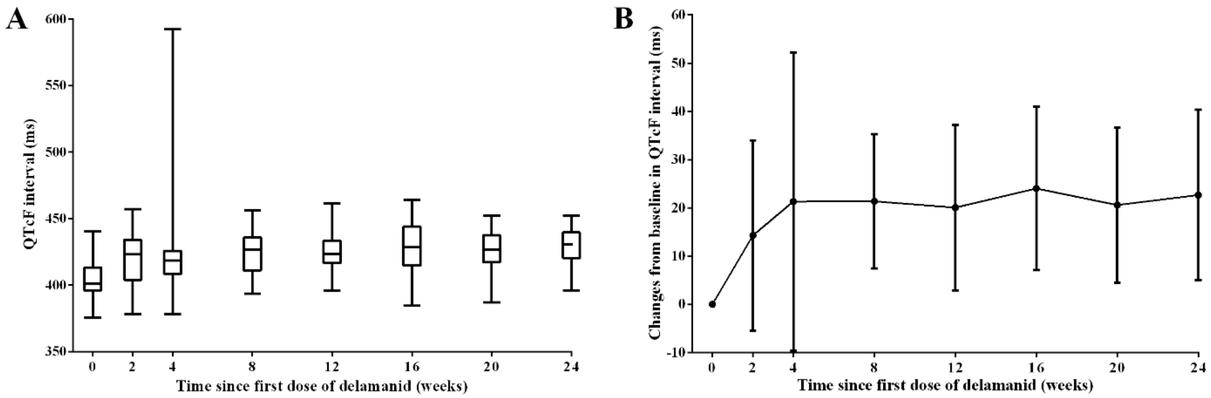


**Supplementary Figure 1. (A) Box plot of QTcF interval at each treatment point and (B) changes from baseline in the QTcF interval over 24 weeks.**

Abbreviations: MDR, multidrug resistant; QTcF, QT interval corrected using Fridericia's method; RR, rifampicin resistant; TB, tuberculosis
